# Beyond the current knowledge on sarcopenia: new insight on neuromuscular factors

**DOI:** 10.1007/s40520-022-02082-3

**Published:** 2022-02-14

**Authors:** Massimo Venturelli, Carlo Reggiani, Federico Schena

**Affiliations:** 1 Department of Neurosciences, Biomedicine and Movement Sciences, University of Verona, Via Casorati 43, 37131 Verona, Italy; 2 Division of Geriatrics, Department of Internal Medicine, University of Utah School of Medicine, Salt Lake City, Utah, USA; 3 Department of Biomedical Sciences, University of Padua, Padua, Italy; 4 Science and Research Center, ZRS, Koper, Slovenia

## Introduction

In the last decades, one of the fastest growing segments of the human population is comprised of people who are beyond 75 years of age. Unfortunately, not all the elderly are healthy, and among the several age-related dysfunctions that naturally characterize this part of human life, sarcopenia is one of the most devastating. Indeed, understanding the changes in muscle function with advancing age is complicated by the interplay between several factors such as the reduction in muscle mass, altered fiber architecture, and alteration of the skeletal muscle innervation. It is important to note, that several factors interact with the aging process and perhaps exacerbate the age-related sarcopenia, including genetic background, physical activity level, nutritional status, and inflammatory levels.

The current most accepted definition of sarcopenia, updated by the European Working Group on Sarcopenia in Older People (EWGSOP) on the 2019 [1], reports that low muscle strength is the primary sign of this pathology and muscle strength is the most reliable measure of muscle function. A sarcopenia diagnosis is confirmed by the presence of low muscle quantity or quality. When low muscle strength, low muscle quantity and quality and low physical performance are all detected, sarcopenia is considered severe. However, from a physiological point of view, the factors that contribute to this drop in muscle strength and mass are not well defined and specific focus on the neuromuscular causes is imperative.

## Aging and sarcopenia: convergences and divergences

Muscle atrophy is one of the most visible hallmarks of skeletal muscle aging [2–4]. Starting from the beginning of the 4th decade, muscle mass decreases by approximately 0.5% every year [5]. The multifactorial determinants of this phenomena include reduced levels of anabolic hormones, chronic inflammation, degradation of the muscle contractile proteins, loss of regenerative capacity, altered neural activation, and mitochondrial dysfunction [5, 6]. The drop of skeletal muscle mass implicates a loss of contractile function, originating a decrease of force and power. However, physiological studies show a dissociation between the changes in skeletal muscle volume and the related decrease in voluntary force in older adults, clearly documented when maximal voluntary contraction (MVC) normalized to muscle physiological cross sectional area. This divergence between the skeletal muscle atrophy and the more pronounced reduction in muscle voluntary force strongly suggests that additional physiological mechanisms contributing to the recognized age-related sarcopenia. Among those mechanism a primary role is played by alterations of the neural control, and will be discussed in this viewpoint article [7].

## Neuromuscular factors interested in the age-related sarcopenia

**Fig. 1:**
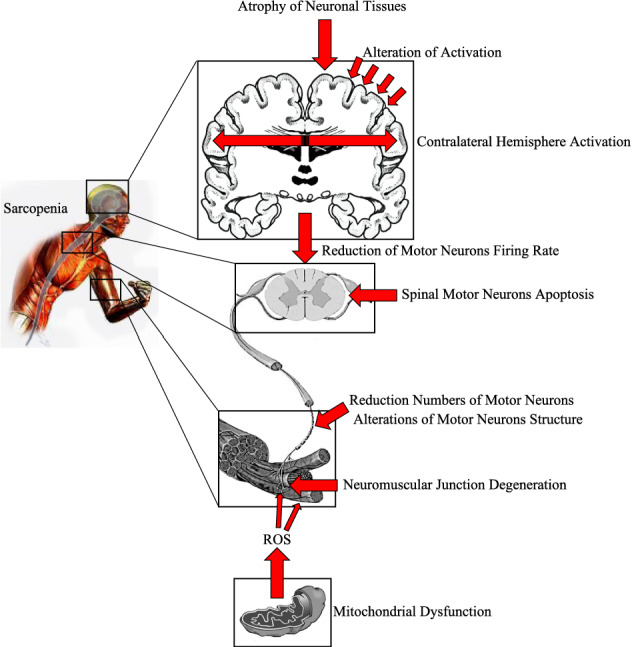
Conceptual schematic of the neuromuscular factors responsible for age-related sarcopenia. The reduction in cortical inputs, increased cerebral cortex atrophy, increased motor-neurons apoptosis and neuromuscular junction impairment associated with increased levels of reactive oxygen species (ROS) derived from mitochondrial dysfunction are key factors underpinning the loss of skeletal muscle mass and function

From a physiological point of view, voluntary force represents the integration of cortical inputs to motor neurons, motor neuron discharge, neuromuscular junction transmission, and the muscle contractile response [8]. It is well-established that potential candidates for the recognized age-related decline in voluntary force include the reduction in the number of motor neurons, alterations in motor unit structure and function, and reduced motoneuron firing rate, causing an attenuated skeletal muscle contractility (Fig. 1) [9]. The available evidence supports the view that the alterations of the cortical inputs to motor neurons are associated with the age-related changes of the cortical neurons. Indeed, there is an atrophy of neuronal tissues in the central nervous system [10], coupled with a considerable reduction in the number motor cortex cells, associated with a decrease of gray and white matter volumes. Indeed, the decline of specific motor-task performance is correlated with the reduction in cerebral cortex thickness. Moreover, during aging the activity of the cerebral cortex is characterized by alterations in the balance between inhibition and activation processes, in different cortical and sub-cortical areas. For instance, it has been showed in elderly that there is a decreased inter-hemispheric inhibition [10], shorter silent period [11], and a reduced intra-cortical inhibition after fatiguing exercise (Fig. 1). It is also interesting to note that the contralateral hemisphere is heavily activated during motor tasks in the elderly [12]. This lack of cortical inhibition areas may lead to hyper activation of additional motor units. Whether these changes in cortical inputs are the result of a natural degradation of neural functions or are compensatory mechanisms, is still a matter of debate [10].

Indeed, aging is also paralleled by changes in motor unit morphology and function, combined with distorted neural activity at spinal and supraspinal levels [13]. Evidence shows that the age-related apoptosis of spinal motor neurons appears relatively earlier in relation to the decline in human muscle mass [13]. It is important, however, to underline that the loss of motor units becomes relevant after the 7th decade of life. The etiology of these age-related morphological changes in motor neurons can be likely found in the accumulation of oxidative stress [13]. Coupled with this rarefaction of the motor neurons, there is limited or partial reinnervation of the denervated muscle fibers by adjacent axons through collateral sprouting [13]. This leads to a lower number of larger motor units, implicating dramatic consequences in the force production [14]. In addition, aging is also coupled with substantial remodeling of the neuromuscular junction [15] that further decreases motor unit activation (Fig. 1). In details, this phenomenon can be triggered by the age-related accumulation of reactive oxygen species (ROS) impacting motoneurons structure, with a consequent fragmentation of the neuromuscular junction. From a different point of view, this phenomenon can be triggered by the mitochondrial over production of ROS in the skeletal muscle fiber, causing a degeneration of the neuromuscular junction and a consequent death of the motoneuron because of lacking of neural activation. Another hypothesis is that neuromuscular junction is impacted by aberrant neural activity of the central motor drive that is reduced during aging. Therefore, sarcopenia likely represents the interplay of different mechanisms with differing time courses and etiologies during the human lifespan [7].

The effectiveness of voluntary activation of motor units and skeletal muscle recruitment is another critical aspect that needs to be accounted for. The literature on this matter is disparate, some studies report lower activation levels in the elderly, while others indicate preserved levels of activation [16]. In this scenario, Venturelli et al. [17] found that the contribution of voluntary activation is a major modulator of the decline in voluntary force production in oldest-old individuals. Specifically, despite a progressive decline in voluntary force generation, the locomotor limbs of the elderly showed a similar reduction in voluntary activation independent from the preservation of locomotion. This decline in muscle voluntary activation was also apparent in upper limbs. Further proof of the potential neuromuscular contribution to the age-related sarcopenia is provided by the determination of force generated by a single supramaximal electrical stimulus normalized to muscle cross sectional area (electrical evoked resting twitch). This variable is an indicator of contractile force, without the neural drive contribution, and evidence showed a preservation of resting twitch specific force in the locomotor and non-locomotor limbs of elderly [17]. As reported in the schematic Fig. 1, it seems reasonable to assume that a deficit in neural drive plays a significant role in the clinical manifestation of age-related sarcopenia.
